# A Simple and Efficient Substitute for the Stomach Pump

**Published:** 1870-09

**Authors:** 


### A Simple and Efficient Substitute for the Stomach Pump.

Prof. John T. Hodgen states (St. Louis Med. and Surg. Journ., July, 1870) that about a year ago he had a case of stricture of the œsophagus, so narrow that his patient could not swallow even liquids. "To sustain life I resorted to a small stomach tube (a gum catheter, in fact); as a means of injecting liquid nourishment; to this I fixed the elastic tube of one of Davidson's syringes.

On one occasion the vessel containing the liquid happened to be higher than the patient's stomach, and I observed while the syringe was not being used, that the liquid continued to flow into the stomach—the action being that of a syphon. I at once, to test the syphon, substituted a simple elastic tube for the syringe, and found the stomach could be as readily emptied as filled. Thus I conceived the idea of using a syphon instead of a stomach pump, and have used the same in a case of poisoning, recently, with the most complete success.

I attach four feet of india-rubber tubing to a stomach tube, fill both with water, by simply dipping it into the liquid, end first, then compressing the elastic tube between the thumb and finger, to keep the fluid from running out; introduce the stomach tube; lower the outer end of the elastic tube, and the contents of the stomach pour out as readily as if from an open vessel. When the fluid ceases to flow, I dip the outer end of the tube beneath the surface of water, elevate the vessel containing it, and the stomach is soon filled; lower again the outer end of the tube, and the stomach is emptied. This can, of course, be repeated as often as is necessary.

The advantages claimed for this simple contrivance are, that it may be almost always improvised, is of speedy and easy application, has no valves to become obstructed or deranged, and is less expensive than a stomach pump.

The same principle may be applied in injecting fluid into the bowels, as indeed it has been for injecting into the bladder, uterus, and vagina."—*Medical News.*

